# Alfred Otto Carl Nier: On the Shoulders of a Mass Spectrometry Giant

**DOI:** 10.1021/jasms.4c00193

**Published:** 2024-07-12

**Authors:** Francisco José Díaz-Galiano

**Affiliations:** University of Almería, Department of Chemistry and Physics, Agrifood Campus of International Excellence (ceiA3), Ctra. Sacramento s/n, La Cañada de San Urbano, 04120 Almería, Spain

## Abstract

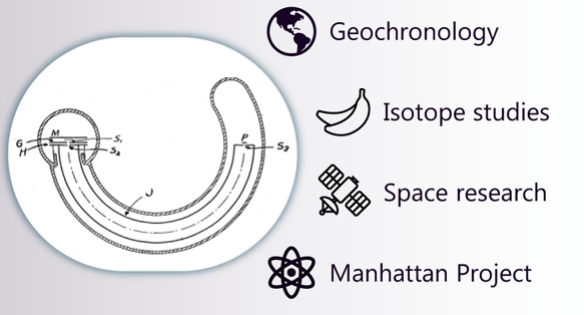

This Perspective pays homage to Alfred Otto Carl Nier, whose substantial contributions were fundamental in shaping the mass spectrometry field into a key technology in research and industry. On the 30th anniversary of his passing, on May 16, 1994, this paper explores Nier’s role in the field of mass spectrometry through an overview of his published works, key interviews, and archival material. Nier, originally an electrical engineer turned physicist, spent most of his scientific career at the University of Minnesota. His many innovations, both instrumental and methodological, encompassed advanced fields such as isotopic research, tracer studies, geochronology, or space research. Nier improved sector mass spectrometers, participated in the development of the isotope-ratio mass spectrometry field, developed a double-focusing sector mass spectrometer, and was a relevant member of the Manhattan Project. Today, Nier’s influence persists, inspiring new generations of scientists engaged in cutting-edge research, from environmental studies to planetary exploration. His legacy thrives as current technologies and scientific strategies still echo his innovations and foresight.

## Alfred Otto Carl Nier

It is not an overstatement to affirm that the evolution of mass spectrometry into the robust research and industrial tool it is today is, in large measure, attributable to Alfred Otto Carl Nier. Nier, an electrical engineer turned physicist, came to the field of mass spectrometry somewhat serendipitously under the direction of John T. Tate at the University of Minnesota, in the United States (US). One of his most enduring contributions to the field of mass spectrometry is the Nier ion source, which has remained the standard for producing gas-phase ions via electron ionization (EI) in gas chromatography–mass spectrometry (GC-MS) instruments for 84 years since its design in 1940.^1^ As we approach the 30th anniversary of his death on May 16, 1994, the purpose of this paper is to serve as a tribute to the contributions of Alfred O. Nier—as he commonly signed his scientific publications—to provide a short overview on his extensive work, to contemplate on his legacy, and to introduce new researchers to his figure.

Throughout the years, the aspects of Alfred O. C. Nier’s varied career have been discussed in the literature by authors such as Michael A. Grayson, archivist for the American Society for Mass Spectrometry (ASMS),^2,3^ Scolman and Johnson,^4^ Steven J. Pachuta,^5^ John de Laeter,^6,7^ and even by his own son, Keith A. Nier.^8,9^ Alfred O. C. Nier himself also devoted his later years to the reminiscing of his scientific career path, both in the form of manuscripts and through interviews.^10−16^ In particular, the four-day-long interview conducted by Thomas Krick and Michael A. Grayson in April of 1989 stands out as an invaluable source of insight into the figure of Alfred O. C. Nier and the development of the mass spectrometry field, and is a must-read for any researcher keen on learning about this topic.^14^ As one can learn from reading through these works and those of others with whom he collaborated, Al Nier—as those who knew him referred to him—is considered the greatest contributor to modern mass spectrometry not only for his inventions but also for assisting anyone interested in implementing his technology.^6^

## Nier’s Introduction to Mass Spectrometry

**Figure 1:**
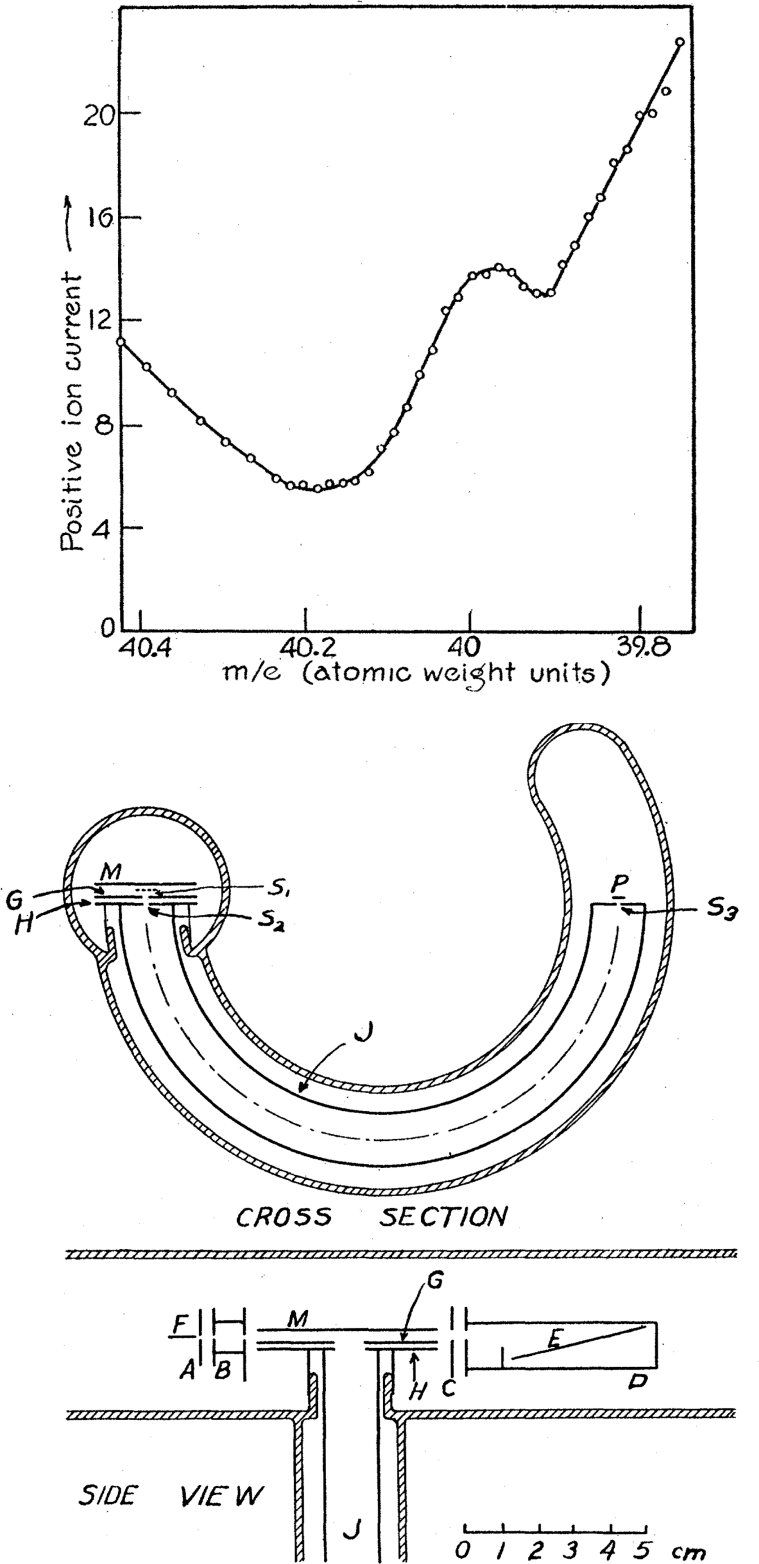
(Top) First mass spectrum confirming the existence of ^40^K. (Bottom) Scheme of Alfred O. C. Nier’s first mass spectrometer. Reproduced from the work by Nier (1935 and 1936) with permission from the American Physical Society.^21,22^

Initially, Alfred O. C. Nier’s work was focused on electrical discharges in gases and EI phenomena. Later, Prof. Tate suggested that Nier begin working on the development of a mass spectrometer with a new hire in the laboratory. Before long, Nier found himself working independently on mass spectrometry in Tate’s laboratory, with his first article on the subject being published in 1935.^2,12,17^ The mass spectrometer designed by Nier was itself an evolution of Bleakney’s and Tate and Smith’s designs. Nier’s version contained a device which compensated for fluctuations in the magnetic field by automatically adjusting the electric field of the instrument. The resulting ion trajectories were stable, as the ions’ deflections became largely independent of the fluctuation of the magnetic fields:^17−19^ [*m*/*z*] = [*r*^2^*H*^2^(2*E*)^−1^], where *m*/*z* is the mass-to-charge ratio, *r* is the curvature radius, *H* is the magnetic field strength, and *E* is the accelerating voltage. When the magnetic field fluctuates, in turn, the *m*/*z* for which the instrument is configured also changes. Since this *m*/*z* is proportional to *H*^2^/*E*, automatically adjusting *E* according to *H* fluctuations allowed the instrument to stay in focus for the configured *m*/*z*.^17^ In that same year, using an improved version of the instrument with further refinements based on Distad and Williams’s work, Nier reported the existence of ^40^K for the first time (Figure 1).^20−22^ This discovery was met with some controversy, as Keith Brewer, a physicist studying isotopes, submitted a manuscript to the same journal as Nier at the same time, wherein he stated that ^40^K did not exist at the abundance levels reported by Nier. John Tate, the editor of the journal, asked Brewer to rerun his experiments, which he did, resulting in the confirmation of Nier’s discovery.^2^ The significance of Nier’s finding would soon be established. Shortly thereafter, Nier studied the isotopic composition of several other elements, including Rb, Zn, Cd, and Ar.^22^

## Nuclide Charting Using Mass Spectrometry

By 1936, Alfred O. Nier’s expertise was unsurprisingly in high demand. In that year, he joined Bainbridge’s research team at Harvard, where he built yet another improved mass spectrometer. With this, he determined the isotopic constitution of several more elements such as Os, Hg, Xe, Kr, As, Ba, Bi, and Sr.^2,23−25^ The resolving power (*R*)—a measure of the spread of peaks in a spectrum—this instrument offered was about *R* = 500 for an *m*/*z* of 192, measured at full-width half-maximum (fwhm), while his 1936 instrument’s resolving power was approximately *R* = 170 for an *m*/*z* of 36 (with *R* = [*m*/*z*][Δ*m*/*z*]^−1^).^22,23^

Nier’s 1937 mass spectrometer, in lieu of using a solenoid, was operated between the poles of an electromagnet. This new iteration was roughly three times as large as his 1936 design, resulting in greater resolving power with equivalent sensitivity.^24^ However, it was inferior in terms of the resolving power and sensitivity of contemporary mass spectrographs, such as those built by Bainbridge and Mattauch and Herzog.^3,26^ Mattauch and Herzog, who had begun working on a new mass spectrograph in 1932, are credited with developing the first double-focusing mass spectrograph. This significant development greatly enhanced the resolving power and sensitivity of previous devices, and it is known as the Mattauch–Herzog geometry.^26,27^ The two researchers, Nier and Mattauch, would later become both rivals and friends. A notable collaboration was their campaign for the standardization of atomic masses relative to ^12^C = 12 Da in the 1950s, replacing the O = 16 Da used by the International Union of Pure and Applied Chemistry (IUPAC) and the ^16^O = 16 Da used by the International Union of Pure and Applied Physics (IUPAP).^3,28^

Returning to Nier’s work in the 1930s, his research on isotopes demonstrated the capabilities of his mass spectrometer not only to detect the presence of isotopes for many elements, but also to accurately measure the isotopic abundance of each species. A year after Nier discovered ^40^K and accurately measured its abundance at 1/8600 relative to ^39^K, Bramley reported the radioactive decay of ^40^K into ^40^Ar.^29^ The ^40^Ar isotope, the third most-abundant gas in Earth’s atmosphere, is the result of ^40^K decay. Conversely, ^36^Ar is the most common isotope in interstellar media, not ^40^Ar.^30^ Later on, Nier would collaborate with L. T. Aldrich to hypothesize that comparing the ^40^Ar/^36^Ar ratio in rocks and in the atmosphere could provide a method for geological dating, now known as K–Ar dating.^31^ The K–Ar dating method was confirmed within the next decade and is commonly used today.^12,32,33^ The discovery of ^40^K was undoubtedly significant for a scientist’s second publication. Nier also provided critical insight into the future field of geochronology by measuring the relative abundances of lead isotopes with his 1937 mass spectrometer. In this context, Nier carried out research to determine the processes by which uranium and thorium decay into lead.^34^ If a rock or mineral specimen is originally free of lead, since the decay rates of uranium into lead and thorium into lead are known, it is possible to determine the age of said specimen by measuring the ratio of the radioactive uranium or thorium isotopes and the daughter lead isotopes (among several other approaches). In the case of uranium, ^238^U decays into ^206^Pb while ^235^U decays into ^207^Pb, and in the case of Th, ^232^Th decays into ^208^Pb.^3^ However, since ^204^Pb is a primordial nuclide, and as there are always varying amounts of lead present in every sample, the relative abundance of this isotope compared to the radiogenic nuclides can also serve as a basis for dating.^3,35,36^ Consequently, Nier’s mass spectrometric method for determining the isotopic abundances of lead proved vastly superior to the available wet chemistry methods. Nier’s results were so convincing that even a Harvard professor, once a staunch defender of these wet chemistry methods, was captivated by mass spectrometry analyses and became Nier’s assistant in preparing the numerous samples that came into Nier’s hands:^34^

“*The writer wishes to express his appreciation to Professor G. P. Baxter of the Department of Chemistry, Harvard University, who very kindly prepared and furnished 11 of the 12 samples used. It was only through his interest and cooperation that this work was possible*.”—Alfred O. C. Nier, 1938.^34^

Nier, of course, benefited from Baxter’s vast expertise in the field: Baxter was the successor of Nobel Prize-winner Theodore Richards, developer of a chemical method to determine atomic weights.^6^

Alfred O. C. Nier returned to the University of Minnesota in 1938 to care for his parents. At his return to Minnesota, he divided his attention between mass spectrometry and thermal diffusion. Nier had been working on the measurement of the relative abundances of ^13^C and ^12^C in several materials at the same time he studied the uranium isotopes while in Harvard.^37^ With thermal diffusion, it is possible to obtain gases (e.g., methane) enriched in a given isotope, since the technique permits the fractionation of gases of different molecular weight. Then, the enriched gas can be used as a building block for more complex substances which differ in the isotopic ratio compared to naturally occurring substances. Nier’s mass spectrometer allowed the detection of these isotopic differences with remarkable precision and accuracy, allowing for tracer experiments. Nier collaborated with a number of researchers during 1940 and 1941 on these tracer experiments with ^13^C-enriched methane, setting the foundations for yet another scientific discipline. The study of the biosynthesis of kojic acid was one of the first uses of the isotope tracer techniques.^38^ Today, these studies continue to be performed similarly to those in which Nier participated and are also commonly employed in medical research.^2^ One of Nier’s most significant contributions to this field was his collaboration with John Bardeen—awarded twice with the Nobel Prize in Physics—in the construction of a 22.5 m thermal diffusion column which could provide methane with almost 12% of ^13^CH_4_, compared to the 1.109% naturally occurring abundance.^39^

## The Manhattan Project

**Figure 2:**
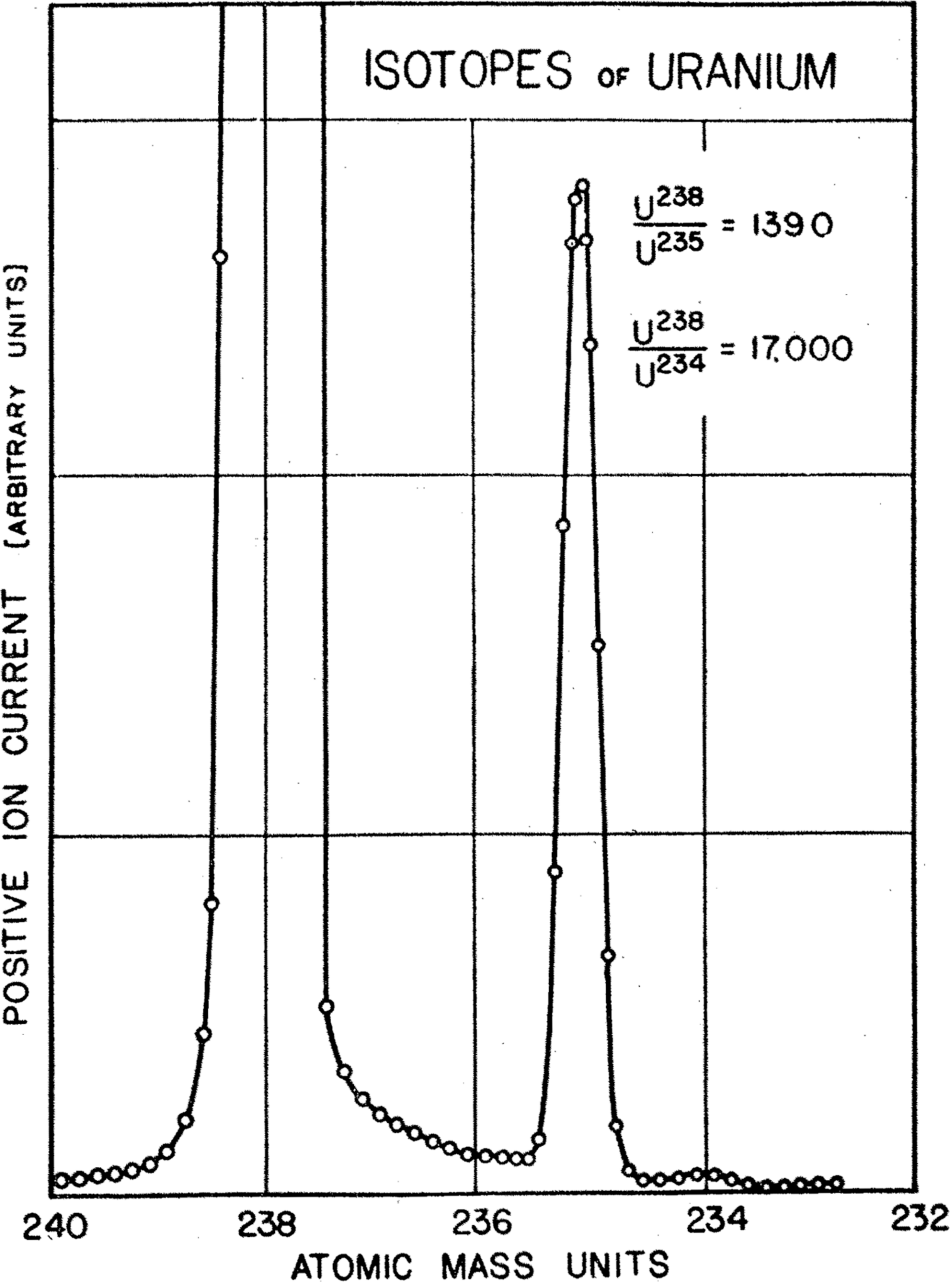
Determination of the relative abundance of ^234^U, ^235^U, and ^238^U. Reproduced from the work by Nier (1939) with permission from the American Physical Society.^40^

Nier’s ^13^C enrichment studies were not, however, his greatest contributions following his return to the University of Minnesota. Researchers had not yet carried out the exact measurement of uranium isotopes’ abundance,^15^ something that Bainbridge suggested Nier could evaluate with his mass spectrometer.^40^ A year after his arrival, in 1939, he met John Dunning and Enrico Fermi, who proposed that he adapt his mass spectrometer to collect separated ^235^U and ^238^U isotopes to determine which of them was the fissionable isotope. For some time, Nier did not pay much heed to Fermi’s request, with his perspective changing upon receiving a persuasive letter from Fermi himself in the autumn of 1939. Nier shared the original letter sent by Fermi in his 1989 recollection of his time in the Manhattan Project, a document the reader is strongly encouraged to access.^11^ However, Nier had been unable to find a way to obtain volatile uranium samples.^3^ It was Baxter who provided Nier with volatile uranium compounds, which he used to determine the isotopic abundances of uranium, reporting the previously unconfirmed ^234^U isotope (Figure 2) discussed by Arthur J. Dempster.^40,41^ Furthermore, between 1939 and 1941, Nier also calculated the half-lives for the three uranium isotopes and established the basis for geochronology measurements. Nier demonstrated the age of the Precambrian and laid the foundation for the calculation of the Earth’s age.^10,36,40,42,43^

**Figure 3:**
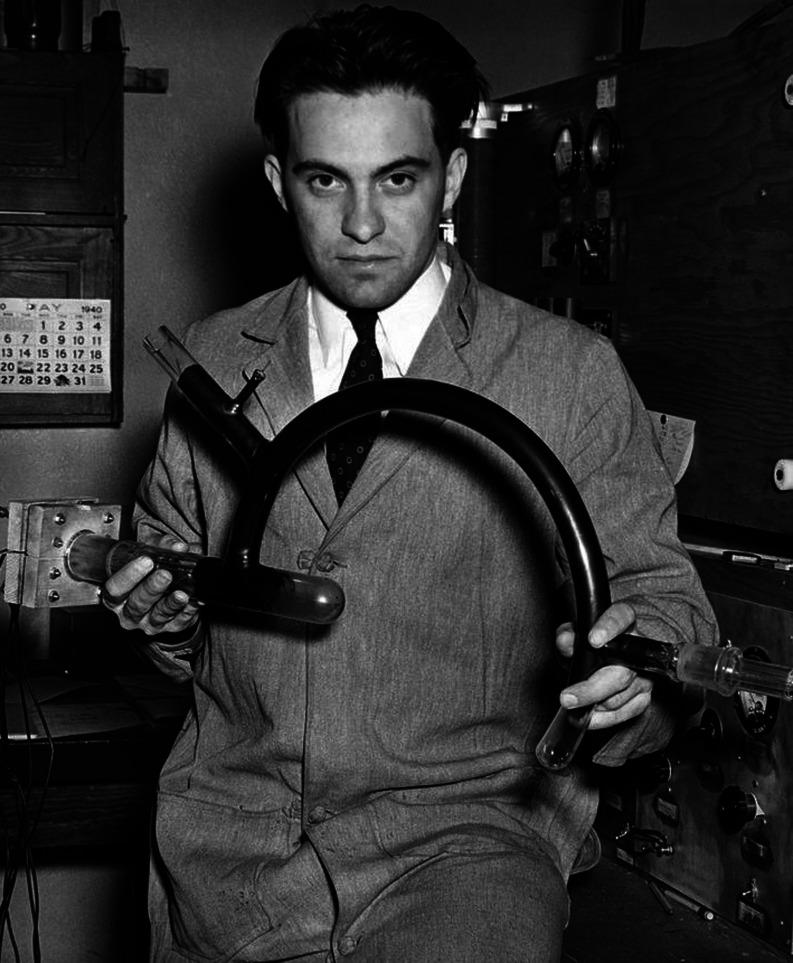
Picture of Alfred O. C. Nier’s 180° sector magnet mass spectrometer, which he used in the experiments on uranium. Reproduced from the work by de Laeter and Kurz (2006) with permission from Wiley.^6^

The modification of the mass spectrometer was not an easy task, but between February 28 and 29, 1940, Nier succeeded in collecting minute amounts of both isotopes from Baxter’s old samples (around 1.5 ng) and sent them to Dunning for testing.^2,6,11^ Shortly afterward, as had been predicted by Niels Bohr and John Wheeler, Nier and Dunning demonstrated that ^235^U was the isotope responsible for slow neutron fission.^44^ Experiments on uranium isotopes continued in 1940, determining that most fast neutron fission activity was due to ^238^U.^45^ The instrument Nier used to isolate the uranium isotopes is shown in Figure 3. As a consequence of these findings, ^235^U enrichment experiments soon commenced in the US, and Nier’s instrument was the only device in the world capable of rapidly evaluating the relative abundance of ^234^U, ^235^U, and ^238^U.^3,11^

**Figure 4:**
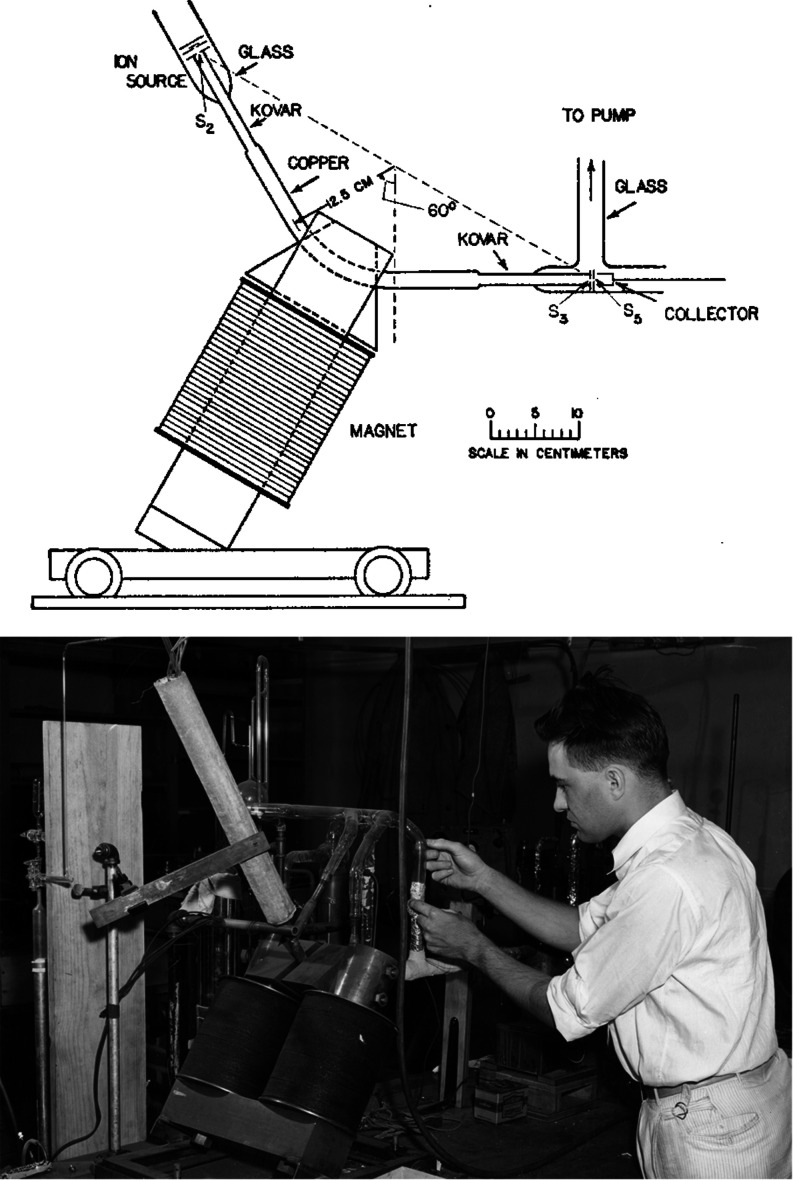
(Top) Scheme and (bottom) picture of Alfred O. C. Nier’s 60° sector magnet mass spectrometer. Scheme reproduced from the work by Nier (1940) with permission from AIP Publishing.^1^ Picture reprinted with permission from Griffiths, J. A Brief History of Mass Spectrometry. *Anal. Chem.***2008**, *80* (15), 5678–5683. Copyright 2008 American Chemical Society.^94^

Unsurprisingly, he was invited to join the Manhattan Project after the attacks on Pearl Harbor.^3^ The required secrecy and commitment to the task would result in no scientific publications between 1942 and 1945. At first, only Nier’s instrument could measure uranium isotopic abundance ratios. By 1942, Nier’s team (which included Mark Inghram, one of Dempster’s students) had built an additional seven instruments capable of this task.^11^ Back in Minnesota, the workload for Nier’s instrument had been immense, considering both the ^13^C and uranium analyses. For this reason, in 1940, he had embarked on the construction of a new mass spectrometer based on a 60° sector magnet for the analysis of isotopic ratios. The size of the required magnets (and the power consumption) was significantly reduced in this new design compared to those of the 180° magnets he had been employing, making them portable (Figure 4).^1,11^ This type of instrument, which could also measure isotopic ratios with great accuracy, was adapted for the needs of the Manhattan Project. Nier’s team built 12 mass spectrometers based on the 60° sector magnet design to determine deuterium concentration in heavy water. The design of these smaller devices became crucial in the separation of uranium isotopes, which involved the gaseous diffusion of UF_6_. However, when in contact with moisture, this compound would clog the diffusion instrument, which rendered the plant useless.^2^ The deuterium detector mass spectrometer was adapted to detect helium leaks (since ^4^He is produced in the uranium and thorium decay chains),^6^ which allowed the operators to check all seals and connections for leaks. This helium detector, a crucial part of the Manhattan Project, was kept secret until Nier et al. described it in a post-World War II (WWII) article.^91^ This was not Nier’s last contribution to the Manhattan Project. Quite the contrary, his team tackled another difficult task in 1943. It was necessary to evaluate the impurities in the process stream, which was cooled with multiple refrigerant units, so Nier developed a mass spectrometer (again, based on the 60° sector magnet design) that could, for the first time, perform the online monitoring of the process stream.^46^ The uranium enrichment plant was divided in 50 buildings, each of them with two online mass spectrometers (one serving as a backup). Before this invention, the process had to be evaluated by taking a sample and evaluating its composition in an external laboratory.^2^ Accounting for the deuterium, helium, and online mass spectrometers, over 100 such devices were used in this facility, one of the largest (if not the largest) such installation attempted.^2,11^ With the success of the Manhattan Project, the scientists involved returned to their regular duties in 1945, Nier included. As stated earlier, Nier himself provided first-hand accounts of his involvement with the Manhattan Project in 1989, which are strongly recommended for the readers.^2,11,14^

## Post-WWII

After WWII, interest in mass spectrometry and its applications greatly increased. This surge of interest was driven largely by the widespread distribution of Nier’s mass spectrometers following the end of the Manhattan Project, which allowed more scientists to become involved in the field. This expanded engagement led to a significant number of innovations between the late 1940s and the 1960s. These innovations included the first time-of-flight (TOF) instrument, which was proposed by W. E. Stephens in a 1946 short communication titled ‘*A Pulsed Mass Spectrometer with Time Dispersion*’. Other significant advancements during this period included the development of the quadrupole mass filter and the quadrupole ion trap—also known as the Paul trap, in honor of Wolfgang Paul.^47−52^ Meanwhile, Nier’s 60° sector magnet mass spectrometer allowed research teams across many fields to use this analytical technique for their needs.^6^ While the precise determination of the masses of the isotopes by mass spectrographs (like those built by Bainbridge or Mattauch) was as yet unrivaled, Nier’s instruments stood out for their unsurpassed ability in terms of relative abundance determinations. His mass spectrometers’ superiority was further established in 1947. That year, Nier presented a 60° sector magnet mass spectrometer with multiple collectors.^53^ Shortly thereafter, he also introduced a method for measuring two small currents with the same relative magnitudes, and in 1948, he described, in collaboration with Rene Bernas, the process by which intense ion beams could be produced in a mass spectrometer.^54,55^ Nier used his 60° sector magnet mass spectrometer to once again determine the relative abundances of the isotopes of C, N, O, Ar, K, Ne, Kr, Rb, Xe, and Hg.^56,57^ Harold C. Urey, for whom Nier had worked on the Manhattan Project, built a modified version of Nier’s latest instrument with his assistance.^58^ This study, alongside the relative ^13^C/^12^C Nier had conducted between 1939 and the start of the Manhattan Project, marked inception of another field pioneered by Nier: isotope-ratio mass spectrometry (IRMS).^3,37^ IRMS is a unique MS technique, first pioneered by Nier and Urey, as previously discussed, that quantifies the relative abundance of isotopes from the same element in a sample.^53,54,58−60^ The majority of IRMS instruments are sector mass spectrometers due to two key reasons: (i) the capability to use multiple collectors and (ii) their ability to provide high-quality peak shapes.^58,61,62^ The relative abundance of stable isotopes is invariably given in terms of the heaviest isotope. This convention, traditionally ascribed to Urey, is referred to as “delta” (δ). Urey used an IRMS instrument constructed with the assistance of Alfred O. C. Nier to measure the relative isotopic abundance of δ^18^O in fossilized calcium carbonate samples in 1948. The aim was to assess whether the oxygen isotopic composition in the ocean had remained stable or varied over millions of years—in other words, to evaluate ocean temperature changes through geological epochs.^11,58^ However, it was Nier, not Urey, who first described the δ^13^C isotopic signature in a 1946 book he coauthored and coedited:

“*There are no hard and fast rules as to the best way to report data obtained in tracer isotope experiments.* [ . . .] [*I*]*t would seem highly advisible to always include in the published results the excess of tracer isotope* [ . . .] *above that found in some arbitrary standard.* [ . . .] *If* [ . . .] *ordinary chemical carbonate is used* [ . . .] *the normal average biological material will probably contain less C*^*13*^*than does the laboratory standard and it and the more dilute samples studied will have a negative excess of C*^*13*^*over the standard. While this may be disconcerting to the reader, in any biological experiment what is really important is the difference in C*^*13*^*concentrations in different compounds*.”—Alfred O. C. Nier, 1946.^63^

For this reason, the milliUrey (mUr) units sometimes associated with IRMS measurements should be milliNiers (mNi) instead.

**Figure 5:**
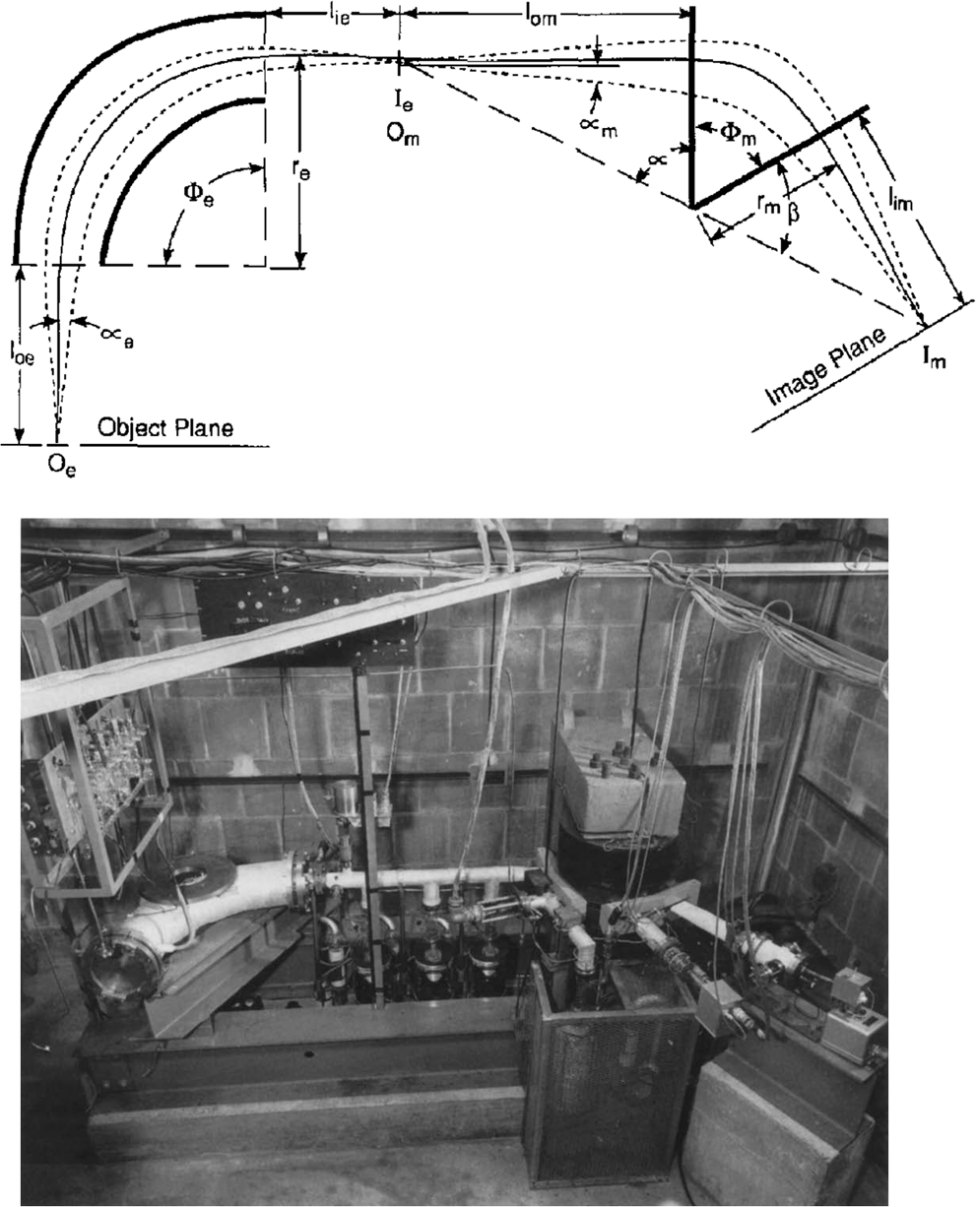
(Top) Scheme and (bottom) picture of Nier–Johnson’s 1953 double-focusing magnet mass spectrometer. The bottom picture is mirrored to match the scheme. Reprinted with permission from Nier, A. O. The Development of a High Resolution Mass Spectrometer: A Reminiscence. *J. Am. Soc. Mass Spectrom*. **1991**, *2* (6), 447–452. Copyright 1991 American Chemical Society.^13^

Soon after, also in 1948, Nier began working on a double-focusing mass spectrometer, a design which until then had been limited to mass spectrographs. He entrusted this task to Edgar Johnson, who was a graduate student at the time. The new instrument achieved atomic mass measurements with unprecedented precision by combining a symmetrical 90° electrostatic analyzer and an asymmetrical 60° magnetic analyzer. The instrument was first reported in 1951, with Johnson detailing the theory behind its operation in 1953.^6,13,64,65^ This double-focusing design became known as the “Nier–Johnson geometry”. The device (Figure 5) offered a resolving power of 600 for an *m*/*z* of 45, enabling it to differentiate CO_2_ (exact mass = 43.9898 Da) from C_3_H_8_ (exact mass = 44.0626 Da). After this impressive accomplishment, Johnson left Nier’s laboratory, never to work again on mass spectrometry.^2^ Venit, vidit, vicit. Nier discussed in detail the development of the instrument, and other high-resolution mass spectrometers, in a 1991 article.^13^ At this time, Nier also found the time to collaborate with researchers from yet another field: the study of metabolic processes in living organisms using mass spectrometry.^66^

## Gazing at the Stars

**Figure 6:**
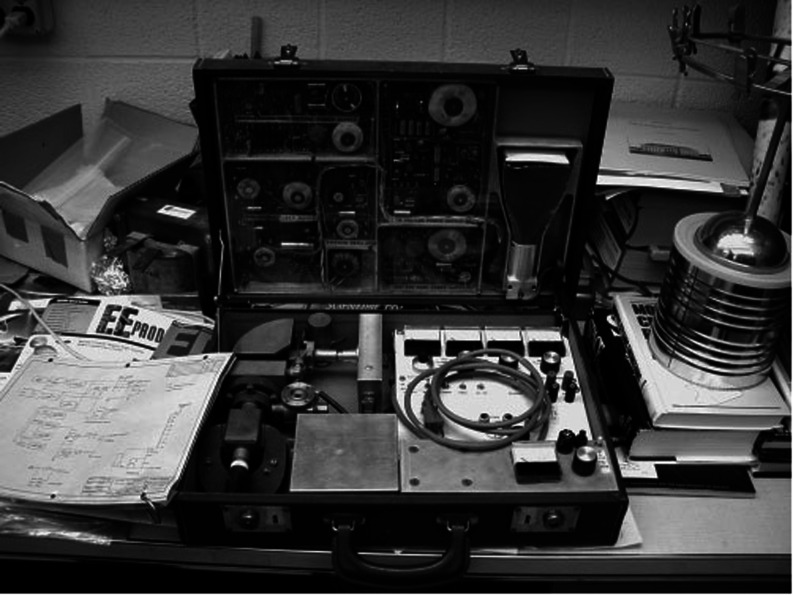
Miniaturized mass spectrometer built by Alfred O. C. Nier in the mid-1960s fit inside a briefcase. Reproduced from the work by de Laeter and Kurz (2006) with permission from Wiley.^6^

**Figure 7:**
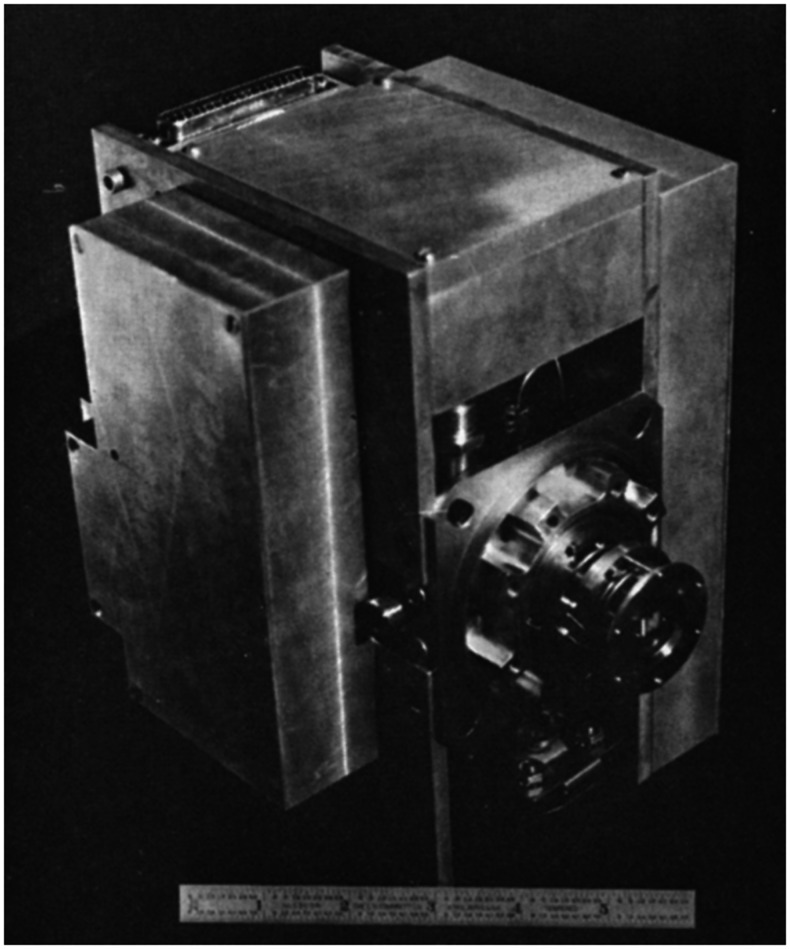
Prototype Mattauch–Herzog double-focusing mass spectrometer built by Alfred O. C. Nier and J. L. Hayden for planetary atmosphere studies. Reproduced from the work by Nier and Hayden (1971) with permission from Elsevier B.V.^69^

Perhaps satisfied with his contributions to geological studies, Nier turned his attention to the skies in the 1960s for his next challenge. To send a mass spectrometer high into the atmosphere or even beyond, size and energy requirements had to be adjusted. In 1960, Nier described a scaled-down version of the Nier–Johnson double-focusing mass spectrometer.^67^ The science behind the smaller mass spectrometer was insufficient to convince National Aeronautics and Space Administration (NASA) officials of the feasibility of including these instruments in spacecraft. To transform the sceptics into believers, Nier built a mass spectrometer that fit inside a regular briefcase, as shown in Figure 6.^6^ NASA officials, now convinced, sent Nier’s spectrometers (a reduced-size double-focusing one and a 90° single-focusing iteration) to a height of 100–200 km in the Earth’s atmosphere to evaluate its composition. Nier et al. determined the content of O, O_2_, N, and N_2_ and their change in abundance with height.^68^ Despite having developed a double-focusing mass spectrometer himself, Nier used the Mattauch–Herzog geometry due to its compactness, the possibility to include more than one ion collector, and its advantage in the detection of low-abundance compounds due to the electron multiplier detectors (Figure 7).^2,5,69,70^

The results sufficiently convinced NASA officials to fit Nier’s instruments into the Viking program, set to explore the Red Planet in the 1970s. The role of the mass spectrometers on Mars was crucial to ascertain whether the team could perform the exobiology experiments they had prepared. Soviet data indicated that argon was present in high quantities in Mars’ atmosphere, which would compromise the results of these experiments. Upon atmospheric entry, all eyes were on the mass spectrometry team, awaiting the outcome. Fortunately, contrary to the Soviet data, Mars’ atmosphere was found to consist mostly of CO_2_.^2,71,72^

The mission was a resounding success, encouraging NASA to continue exploring planetary atmospheres using mass spectrometry, with the involvement of Nier, such as the Pioneer Venus spacecraft, which included both double-focusing and quadrupole mass spectrometers. The mass spectrometric analysis of Venus’ atmosphere revealed unexpected amounts of two noble gases, helium and argon, leading to a reconsideration of the accepted theories about the planet’s interactions with the Sun.^5,73^ A student of Nier’s, John H. Hoffman, was also responsible for constructing a sector mass spectrometer for the analysis of the Moon’s atmosphere as part of the Apollo program.^74^ Today, mass spectrometers continue to play a crucial role in the exploration of the cosmos.^52,75^

Alfred Otto Carl Nier, Al Nier, would not lower his gaze from the stars until his death in 1994.^3,6^

## Legacy

A sentiment often echoed in discussions—though not directly traceable to a specific source—captures the essence of his impact: “*Prof. Nier never stayed long enough in a field to be considered for a Nobel Prize, but nevertheless, he was a key player in many of the research areas that would later be worthy of such an honor*.”

Alfred O. C. Nier developed new low- and high-resolution mass spectrometers, as well as isotope-ratio mass spectrometers, carried out nuclide studies, participated in thermal diffusion studies, developed methods for calculating the age of minerals and, in the process, for the age of the Earth itself, was a key figure in early nuclear science and in the Manhattan Project, collaborated in the study of metabolic processes and tracer methodologies for biological studies, and was a spearhead in the application of mass spectrometry for space sciences, and finally, he devoted his later years to meteoritics as well.^2,4,76^ As Grayson stated after interviewing Nier, “[*T*]*he impact of this one man’s work on* [*the mass spectrometry*] *field is immeasurable*”.^2^

In his lifetime, Alfred O. C. Nier received numerous awards, including the Geological Society of America’s Arthur L. Day Medal, the Geochemical Society’s Viktor M. Goldschmidt Medal, NASA’s Medal for Exceptional Scientific Achievement, the American Chemical Society’s Field and Franklin Award, and the American Geophysical Union’s William Bowie Medal.^76^ Additionally, the University of Minnesota named him Regents Professor of Physics and awarded him an Honorary Doctor of Science title, and he was also honored by the Atomic Energy Commission for his role in the Manhattan Project.^6^ Posthumously, his figure was also recognized: a trigonal silicon nitride, nierite, was named after him, as was Nier crater, on the surface of Mars.^76,77^ There is also a Nier Prize established in 1995 by the Meteoritical Society for researchers under the age of 35,^78^ and the Alfred O. C. Nier Scholarship, awarded by the School of Physics and Astronomy of the University of Minnesota.^79^ Perhaps his best legacy lives on in the research that builds upon his own and in the careers he helped foster. Over the years, Nier was involved in the early stages of renowned scientists, either as a collaborator or as a doctoral advisor. The list includes names such as Edward P. Ney,^54^ who studied cosmic rays, among several different topics, like Nier himself,^80^ the aforementioned John H. Hoffman,^68,74^ and Walter Johnson, whose research continued Nier’s work on atomic mass measurements.^81^

Alfred O. C. Nier was never found too far from a laboratory. On his last day of research, he was working with his assistant Dennis J. Schlutter, with whom he collaborated in the 1980s and 1990s on topics such as the development of a high-performance double-focusing mass spectrometer or the analysis of interplanetary dust particles.^82−84^ Schlutter continued the research he had begun with Nier on interplanetary dust particles until his very recent death, in 2019. Some of the latest research whose origins can be traced back to Nier’s laboratory is the study of samples from NASA’s Stardust mission on comet 81P/Wild 2, on which Schlutter collaborated.^85^ Additionally, space research has also seen in very recent years new mass spectrometers based on the Nier–Johnson geometry, such as the European Space Agency (ESA) Rosetta probe, which was sent to comet 67P/Churyumov-Gerasimenko to investigate its atmospheric isotopes.^70^ This type of mass spectrometer has also been employed in the enrichment of ^81^Kr and ^85^Kr, a similar application to those Alfred O. C. Nier conducted himself.

Many modern IRMS instruments are still based on the original magnetic sector design by Nier and Urey. The applications of IRMS analyses now extend far beyond their initial uses. For instance, IRMS instruments are employed to discriminate between organic and conventional agricultural practices, or in geographical origin traceability experiments.^86,87^ These analyses are also fundamental in differentiating between endogenous and exogenous steroids in antidoping applications in sports.^88^ In line with Nier’s later research interests, IRMS-based analyses of chondrites have provided key evidence for the Giant Impact Theory of lunar origin.^89^ Additionally, they have shed light on primordial water transport phenomena in the solar system and have been crucial in analyzing amino acids in meteorites to search for the origin of life.^89−91^

There is another IRMS device based on a modifier Nier–Johnson double focusing mass spectrometer geometry, the sensitive high mass-resolution ion microprobe (SHRIMP), of which there are a couple dozen such devices around the world and of which John de Laeter was a significant advocate. Instead of the 60° magnetic sector of the Nier–Johnson geometry, it includes a 72.5° magnetic sector in combination with the 90° electric sector.^92^ These instruments, the *descendants* of Nier’s pioneer work, have been critical in dating some of the oldest materials found on Earth.^93^ Moreover, as discussed in the first few lines of this work, his EI source lives on thousands of GC-MS instruments worldwide, unbeknownst to many of their users.^1^ Today, the mass spectrometry technology he helped popularize—from a niche tool to a key component in thousands of laboratories worldwide—continues to shape our understanding of the molecular world, proving indispensable in both routine analyses and groundbreaking research.

On a personal note, I became an admirer of Nier in the process of writing my thesis, as I delved into the history of the mass spectrometry field and learned about his plethora of contributions. I have only become more inspired by him after writing this paper, and I can affirm with certainty that he will continue to be a source of inspiration for new researchers no matter what anniversary we are celebrating. Reading through the literature written by those who had the fortune to meet Alfred O. C. Nier, a quote by Edward Ney on him appears to be an eloquent summary of his figure as a scientist:^76^

“*Al Nier did just about everything that could be done with a mass spectrometer and did it better than most others*.”—Edward Purdy Ney.^76^
